# Supplementary material for: Identification of photoperiod-induced specific miRNAs in the adrenal glands of Sunite sheep (Ovis aries)
Source: Front Vet Sci. 2022 Jul 22;9:888207. doi: 10.3389/fvets.2022.888207 (PMC9354845; doi:10.3389/fvets.2022.888207)
Supplement: Supplementary file 1 [file Data_Sheet_1.ZIP › Supplementary file/miRDeep2 file.zip!/miRDeep2 file/Know_Structure/SPLP42A2/oar-mir-376a.pdf]

miRBase precursor : oar-mir-376a
Total read count : 176
oar-miR-376a-5p read count : 123
oar-miR-376a-3p read count : 46
remaining reads : 7

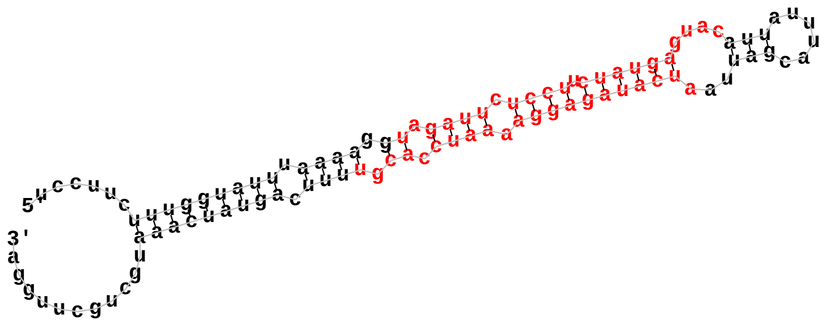

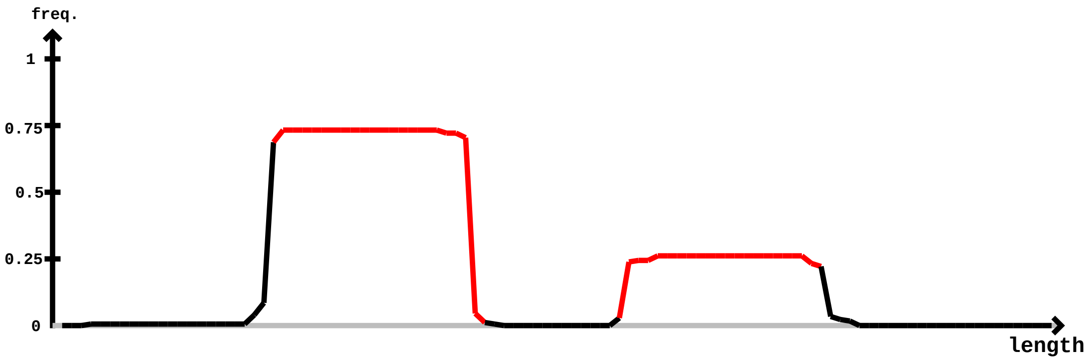

oar-miR-376a-5p

oar-miR-376a-3p

```
5'- uccuucuuugguauuuaaaagguagauucuccuucuaugaguacauuauuuacgauuaaucauagaggaaaauccacguuuucaguaucaaaugcugcuugga  -3'   exp
    ......((((((((.((((.((.((((.((((.((((((....(((......)))...))))))))))).)))).)).)))).))))))))..............  reads   mm   sample
    ..cuucuuugguauuuaaaag..................................................................................  1       0    seq
    ...................agguagauucuccuucuaug................................................................  1       0    seq
    ...................agguagauucuccuucuaugag..............................................................  1       0    seq
    ...................agguagauucuccuucuaugagu.............................................................  4       0    seq
    ....................gguagauucuccuucuaugagu.............................................................  8       0    seq
    .....................guagauucuccuucuaug................................................................  1       0    seq
    .....................guagauucuccuucuaugag..............................................................  2       0    seq
    .....................guagauucuccuucuaugagu.............................................................  99      0    seq
    .....................guagauucuccuucuaugagua............................................................  4       0    seq
    .....................guagauucuccuucuaugaguac...........................................................  1       0    seq
    ......................uagauucuccuucuaugagu.............................................................  5       0    seq
    ......................uagauucuccuucuaugagua............................................................  2       0    seq
    ......................uagauucuccuucuaugaguaca..........................................................  1       0    seq
    .........................................................aaucauagaggaaaauccac..........................  1       0    seq
    .........................................................aaucauagaggaaaauccacgu........................  4       0    seq
    ..........................................................aucauagaggaaaauccac..........................  4       0    seq
    ..........................................................aucauagaggaaaauccacg.........................  2       0    seq
    ..........................................................aucauagaggaaaauccacgu........................  29      0    seq
    ..........................................................aucauagaggaaaauccacguu.......................  2       0    seq
    ...........................................................ucauagaggaaaauccacguu.......................  1       0    seq
    .............................................................auagaggaaaauccacguuuu.....................  3       0    seq
```
